# Health information sources and health‐seeking behaviours of Filipinos living in medically underserved communities: Empirical quantitative research

**DOI:** 10.1002/nop2.2140

**Published:** 2024-03-15

**Authors:** Jennifer Kawi, Miguel Fudolig, Reimund Serafica, Andrew T. Reyes, Francisco Sy, Erwin William A. Leyva, Lorraine S. Evangelista

**Affiliations:** ^1^ School of Nursing, University of Nevada Las Vegas Las Vegas Nevada USA; ^2^ School of Public Health, University of Nevada Las Vegas Las Vegas Nevada USA; ^3^ College of Nursing, University of the Philippines Manila Philippines

**Keywords:** Filipinos, health behaviours, health disparities, health information, health promotion, low‐ and middle‐income countries, underserved

## Abstract

**Aims:**

To describe sources of health information and health‐seeking behaviours of adults (aged ≥18) living in medically underserved communities in the Philippines.

**Design:**

This is a secondary, quantitative analysis from a cross‐sectional parent study. Participants completed a 10‐item, self‐report survey on their sources of health information, healthcare providers sought for health and wellness and health‐seeking behaviours when ill. Responses were evaluated across two age groups (<60 vs. ≥60 years) and genders using generalized linear mixed models.

**Results:**

Surveys were completed by 1202 participants in rural settings (64.6% female, mean age 49.5 ± 17.6). Friends and/or family were their key source of health information (59.6%), followed by traditional media (37%) and healthcare professionals (12.2%). For health promotion, participants went to healthcare professionals (60.9%), informal healthcare providers (17.2%) or others (7.2%). When ill, they visited a healthcare professional 69.1% of the time, self‐medicated (43.9%), prayed (39.5%) or sought treatment from a rural health clinic (31.5%). We also found differences in health‐seeking behaviours based on age and gender.

**Conclusions:**

Our findings highlight the need to organize programs that explicitly deliver accurate health information and adequate care for wellness and illness. Study findings emphasize the importance of integrating family, friends, media and healthcare professionals, including public health nurses, to deliver evidence‐based health information, health promotion and sufficient treatment to medically underserved Filipinos.

**Implications:**

New knowledge provides valuable information to healthcare providers, including public health nurses, in addressing health disparities among medically underserved Filipinos.

**Impact:**

This study addresses the current knowledge gap in a medically vulnerable population. Healthcare professionals are not the primary sources of health information. Approximately one‐third of participants do not seek them for health promotion or treatment even when ill, exacerbating health inequities. More work is necessary to support initiatives in low‐ and middle‐income countries such as the Philippines to reduce health disparities.

**Reporting Method:**

We adhered to the reporting guidelines of STrengthening the Reporting of OBservational studies in Epidemiology (STROBE) for cross‐sectional studies.

**Patient or Public Contribution:**

There was no patient or public contribution as our study design and methodology do not make this necessary.

**Contributions to Wider Global Community**

- Researchers and policymakers need additional data to understand the potential benefits of expanding access to high‐quality health information using modern technology (i.e., the Internet) in rural communities.
- When community health professionals with the appropriate education deliver health education programs, the public may have better access to information on various health topics and advice for improving their health.
- Lack of access to healthcare professionals, healthcare inequalities, lack of investment in healthcare infrastructure and the inaccessibility of clinics in rural areas are all issues that require prompt attention to ensure equitable healthcare for all.

## 1 INTRODUCTION

The 2030 Sustainable Development Goals agenda urges all countries to update and implement their national development policies with an integrated focus on economic, social and environmental development (General Assembly of the United Nations, 2015). This initiative aims to improve the lives of all nations and peoples by 2030, “leaving no one behind.” The primary objective is eradicating poverty and inequality, which affect numerous regions, primarily low‐ and middle‐income countries (LMICs). The promotion of health and wellness is one of the sustainable development goals. It aims to provide a service coverage dimension to ensure that all individuals who benefit from promotive, preventative, curative, rehabilitative or palliative healthcare have access to these services and are of sufficient quality (Bayarsaikhan et al., 2022). For some countries, this would gradually expand the current service coverage and availability level to accommodate epidemiological and demographic changes, such as ageing (The World Bank, 2020). Included are safe, effective, high‐quality and reasonably priced healthcare services (Department of Health, 2020). Yet, the lack of readily available healthcare resources, infrastructure and personnel and inadequate insurance coverage for medical emergencies continue to be obstacles to receiving adequate healthcare in LMICs, including the Philippines. Specifically, the shortage of healthcare resources is exacerbated by the limited understanding of health information sources and health‐seeking behaviours among disadvantaged Filipinos residing in medically underserved areas in the Philippines, which impacts equity in healthcare and limits the goal of “leaving no one behind.”

## 2 BACKGROUND

Over the past few decades, the Philippine population has become healthier. Life expectancy at birth is 70 years, up from 55 years five decades ago. In addition, the number of children who survive to age five has increased. In the 1970s, there were 84 child deaths for every 1000 births. At the start of the century, mortality among children under five was reduced nearly threefold (The World Bank, 2020). These improvements are related to the government's efforts toward universal healthcare (Department of Health, 2020). Yet, the Philippines continues to lag behind other countries in its income bracket in several health outcomes, and considerable provincial and local inequities exist. Moreover, the country receives a low score of 60 out of 100 on the universal health service coverage index, a measure of access to critical services for maternal and child health, infectious conditions and non‐communicable diseases (e.g., heart disease, diabetes, cancer) (World Health Organization, 2022).

Access to credible health information and adequate health‐seeking behaviours are necessary for optimizing health and wellness and managing illness (e.g., initiating care at the proper time and with the appropriate provider; continuing a regular health‐seeking pattern) (Maneze et al., 2015). Seeking out health information or relevant information about one's health through various sources (such as reliable media and evidence‐based printed materials) has long been seen as enhancing patients' understanding of treatment and lifestyle modifications. It is the acquisition of data to clarify or confirm knowledge about a specific subject. Approaches and sources for collecting health information, such as consulting professional healthcare providers, conversing with family/friends and browsing the Internet, can influence the accuracy of information obtained. Individuals with greater knowledge are more likely to maintain control over their condition than those without reliable health information. Moreover, well‐informed individuals are better suited to deal with the uncertainty of disease and the necessary treatments (Teo et al., 2021). Although health information is insufficient to motivate behaviour modification, it is essential and foundational to healthcare.

Over a billion people worldwide, particularly in LMICs, lack access to quality healthcare services (World Health Organization, 2022). The poor performance on access coverage reflects the current state of the country's health system components including service delivery, health financing, human resources, governance and information technology. Likewise, in LMICs, information‐seeking practices and health‐seeking behaviours may differ from those in industrialized countries due to cultural, economic and social variations (Teo et al., 2021). To accommodate for and address the health requirements of individuals in any population, it is crucial to comprehend their health‐ and information‐seeking preferences as these can be reasonably complex. Health information sources and health‐seeking behaviours are important to healthcare, but little is known about these among LMICs, particularly medically underserved populations in the Philippines.

Many barriers exist to accessing and providing healthcare in LMICs like the Philippines. These include a lack of trained professional healthcare providers, inadequate resources for health promotion, a lack of reliable health information sources and the need to seek healthcare advice from various providers (Evangelista & Lorenzo, 2021). Based on data from a report on health and healthcare equity in the Philippines, it is possible to conclude that the poor bear a disproportionate amount of the disease burden and that there are significant disparities in health outcomes between socioeconomic groups (Banaag et al., 2019). A person's propensity to use the healthcare system is influenced by cultural norms and practices and the nature of those norms and practices. Availability, accessibility, affordability and quality of service are also critical, but not always ideal, factors to consider (Maneze et al., 2015). In addition, the individual's socioeconomic status, age, gender, financial resources, how they feel about their health, the nature of their illness and the type of illness are also important considerations (Palafox et al., 2021). As a result, healthcare for individuals residing in medically underserved communities is often substandard because a lack of understanding of their specific needs causes inequities in access to healthcare (Evangelista & Lorenzo, 2021).

While efforts to lessen the health disparities have received constant support from governments worldwide, research on the dynamics and sources of health information and patterns of health‐seeking behaviours among individuals residing in medically underserved communities in LMICs, like the Philippines, is still lacking (Abera Abaerei et al., 2017). For example, there is a paucity of data regarding the information sources most utilized and trusted by individuals from medically underserved communities in LMICs. Likewise, there is a lack of information on the health‐seeking behaviours of this vulnerable population. Lastly, in contrast to the Western setting, where primary healthcare services have been the subject of extensive research, most healthcare systems in LMICs have limited access to and use quality data to help improve care and shape policy (Bitton et al., 2019).

In light of this, our research objectives offer novel information for understanding the sources of health information and health‐seeking behaviours of individuals residing in medically underserved communities in the Philippines with limited access to basic healthcare services. We evaluated health‐seeking preferences for health promotion and behaviours in the context of illness. In addition, we investigated the effects of gender and age on preferred health information sources and health‐seeking behaviours. Gender and age are key variables that are of particular significance among Filipinos given their sociocultural norms and values, particularly in relation to deference to elders and gender roles that influence health and health care (Badana & Andel, 2018; Guerrero, 2022).

In this study among vulnerable Filipinos residing in the Philippines, we define health‐seeking behaviours as the individual's courses of action for health promotion needs and treatment when they are ill. We describe providers as either professional healthcare providers (called *healthcare professionals* from this point) who obtained formal training and education, thereby receiving an academic degree or licensure or *informal healthcare providers* who have no professional education and training, such as faith healers (espiritistas), herbalists (albularyos), quack doctors or massage therapists without formal education (hilots) (Rebuya et al., 2020).

## 3 METHODS

A secondary analysis was conducted using ordinal data from a nationally representative cohort of Filipinos living in medically underserved communities in the Philippines. A convenience sample of 1202 adults ≥18 years old was recruited from rural health clinics in the *barangays* (i.e., villages), with only one adult member in the household eligible to participate. Additional details related to the cross‐sectional, quantitative and descriptive parent study conducted according to ethical guidelines, including informed consent, are described elsewhere (Cacciata et al., 2021). Data specific to sources of health information and health‐seeking behaviours are the foci of this current article. The Institutional Review Boards at a western university in the United States (#REDACTED) and a northern university in the Philippines (Research Ethics Board, #REDACTED) approved this study.

### 3.1 Measures

Tagalog (the local language) and English versions of a 10‐item (excluding demographics), self‐report survey were used for data collection. This is a standardized and validated form developed for the parent study (Cacciata et al., 2021). This measure was adapted from a survey developed specifically for the Filipino community (National Heart Lung and Blood Institute et al., 2003). All the participants chose to complete the questionnaire in English. Trained research assistants were available to help participants respond to the survey when needed. Participants can respond to multiple options when it applied.

#### 3.1.1 Demographics

The survey started with questions on sociodemographic characteristics, including age, gender, marital status, health insurance, education, employment and income.

#### 3.1.2 Sources of health information

Participants were asked where they found and received helpful health and wellness information. Options included friends and/or family, providers, traditional media such as radio or television and print media such as the local English paper, native‐language newspaper or magazines, flyers or community outreach materials and brochures or educational materials in their doctor's office. Participants rated their options from 1 (most often) to 4 (never).

#### 3.1.3 Provider sought for health promotion

Participants were asked whom they go to for their health and wellness checks. Options were either healthcare professionals (e.g., physicians, nurses, acupuncturists), informal healthcare providers (e.g., herbalists, quack doctors) or others (participants were advised to specify).

#### 3.1.4 Health‐Seeking Behaviours for illness treatment

Participants were asked about what they do to treat themselves when they are sick or ill. Options included self‐medicating (i.e., herbal medicines), using medications or home remedies from friends, going to a doctor, seeking traditional healers, praying, going to a rural health centre, seeking advice from a trusted person or nothing. Participants were also advised to check all that applied to them.

### 3.2 Data analyses

Comparisons were conducted on demographics, sources of health information, providers sought for health promotion and health‐seeking behaviours when ill by young and older groups and by male and female participants. Data were analysed and presented, based on the variable measured, as means, standard deviations, ranges, percentages and odds ratios, with a 95% confidence interval.

A generalized linear mixed model approach using the binomial distribution, with random effect to account for variability between each participant, was used to estimate the probabilities for the data on seeking providers for health promotion and treatment for illness. In addition, the Tukey–Kramer adjustment was implemented for multiple comparisons, i.e., between health‐seeking behaviours for illness. Data were analysed using the GLIMMIX procedure in SAS/STAT software, Version 9.4 of the SAS System for Windows. Copyright © (2016) SAS Institute Inc. SAS and all other SAS Institute Inc. product or service names are registered trademarks or trademarks of SAS Institute Inc. (SAS Institute Inc, 2016). A significance level of 0.05 was used in the statistical tests performed in these exploratory analyses.

## 4 RESULTS

Table 1 displays the demographic and social make‐up of the sample (*N* = 1202) according to age group (younger Filipinos were those 18–59 years of age [65.7%] and older Filipinos were those 60 and above [34.3%]). The mean age was 49.5 ± 17.6 years (range 18–83). The majority of participants were female (64.6%), married (58.6%), without insurance (92.6%), high school graduates or higher (96%), with an average annual income less than or equal to PHP 75,000 (58%) and unemployed (51%). Any missing data were not accounted for in the means for the sociodemographics. There were no missing data with respect to the other variables.

**TABLE 1:** Sociodemographic characteristics of the participants (*N* = 1202).

Demographic	Younger Filipinos (ages 18–59)		Older Filipinos (age ≥60)		Total	
	*N*	%	*N*	%	*N*	%
Marital status						
Single	244	30.93	29	7.06	273	22.75
Married	484	61.34	219	53.28	703	58.58
Separated	22	2.79	7	24.14	29	2.42
Widowed	37	4.69	156	37.96	193	16.08
Co‐habitate	2	0.25	0	0	2	0.17
Education Level						
Did not finish High School	8	1.01	40	9.71	48	3.99
High School Graduate or Above	782	98.99	372	90.29	1154	96.01
Insurance						
Yes	72	11.84	2	0.51	74	7.39
No	536	88.16	391	99.49	927	92.61
Gender						
Male	279	35.32	147	35.68	426	35.44
Female	511	64.68	265	64.32	776	64.56
Employment						
Employed	503	85.98	82	14.02	585	48.67
Unemployed	287	46.52	330	53.48	617	51.33
Annual Income						
No income	0	0.00	3	0.76	3	0.30
<PHP 25,000	52	8.57	69	17.56	121	12.10
PHP 25,000–PHP 50,000	169	27.84	145	36.90	314	31.40
PHP 50,000–PHP 75,000	63	10.38	75	19.08	138	13.80
>PHP 75,001	323	53.21	101	25.70	424	42.40

### 4.1 Sources of health information

Table 2 shows a summary of the responses given by the participants on where they get their health‐related information. Among all participants, friends and/or family were the most common source of health information (59.6%), followed by traditional media such as television and radio (37%), print media (18.3%) and healthcare professionals (12.2%). Table 3 shows the frequency of these responses according to gender and age group. There were no significant differences in information sources between men and women. However, a significant interaction was found between information source and age group (*p* < 0.0001). For example, (a) information from television is as likely to be used by both age groups and is significantly more preferred compared to other media (*p* < 0.001) and (b) younger people are more likely to receive healthcare information from healthcare professionals (*p* < 0.001) and the radio (*p* = 0.03) compared to the older people.

**TABLE 2:** Sources of information used most often (*N* = 1202).

Source of information	Total	
	*N*	%
Friends/Family	714	59.6
Healthcare Professionals	147	12.2
TV/Radio	443	37.0
Print Media	220	18.3

**TABLE 3:** Frequency of responses on sources of information according to gender and age group.

Source of information	Younger Filipinos (ages 18–59)	Older Filipinos (age ≥60)
Friends/Family**		
Male	1.83 (1.06)	1.73 (2.30)
Female	1.77 (1.09)	1.40 (0.77)
Healthcare Professionals**		
Male	2.70 (1.03)	2.86 (1.10)
Female	2.57 (0.99)	2.85 (1.05)
Television		
Male	2.31 (1.75)	2.21 (0.78)
Female	2.21 (0.84)	2.41 (2.55)
Radio*		
Male	2.52 (0.97)	2.83 (0.98)
Female	2.75 (2.04)	2.76 (1.00)
Local English Newspapers**		
Male	2.92 (0.86)	3.35 (3.24)
Female	2.88 (1.02)	3.18 (0.94)
Native Language Newspaper**		
Male	3.02 (0.89)	3.22 (0.84)
Female	2.96 (0.92)	3.25 (0.92)
Flyers		
Male	3.06 (0.93)	3.03 (0.97)
Female	2.81 (0.94)	3.02 (1.01)

*Note*: Mean Scores (1‐ most often, 4‐ never). Standard Deviation in parenthesis. (*) denotes a significant difference between younger and older Filipinos at the 0.05 level while a (**) denotes a significant difference at the 0.01 level.

### 4.2 Provider sought for health promotion

Tables 4 presents a summary of the responses regarding the providers sought to promote health. Participants indicated that they sought healthcare for wellness advice and support mostly from healthcare professionals (60.9%), followed by informal healthcare providers (17.2%) or others (e.g., a combination of both providers) at 7.2%. Table 5 shows the frequency of these responses according to gender and age group. The three‐way interactions (provider, age group, gender) and all two‐way interactions, e.g., age group and gender (*p* = 0.8958), gender and provider (*p* = 0.9399), and age group and provider (*p* = 0.3218) were not significant. However, there was a significant overall effect on the type of provider sought for health promotion (healthcare professionals vs. informal healthcare providers, *p* < 0.0001). Overall, Filipinos tend to go to healthcare professionals over informal healthcare providers. Specifically, a Filipino from this sample is estimated to be 27.43 (95% CI: 19.55, 38.48) times more likely to go to a doctor, nurse or acupuncturist than an herbalist or a quack doctor.

**TABLE 4:** Providers sought for health promotion (*N* = 1202).

Health promotion provider	Total	
	*N*	%
Formal Healthcare Professionals	732	60.90
Informal Healthcare Professionals	207	17.22
Both Formal and Informal	87	7.24

**TABLE 5:** Frequency of responses on providers sought for health promotion according to gender and age group.

Health promotion provider	Younger Filipinos (ages 18–59)		Older Filipinos (age ≥ 60)		Total	
	*N*	%	*N*	%	*N*	%
Formal healthcare professionals						
Male	180	64.5	63	42.9	243	57.0
Female	351	68.7	138	52.1	489	63.0
Informal Healthcare Professionals						
Male	53	19.0	20	13.6	73	17.1
Female	91	17.8	43	16.2	134	17.2
Both Formal and Informal						
Male	24	8.6	5	3.4	29	6.81
Female	42	8.2	16	6.0	58	7.47

### 4.3 Health‐seeking behaviours for illness treatment

Table 6 displays a summary of how the participants responded when asked about how they treat their illnesses. When sick, 69.1% primarily saw a doctor. Participants also reported using home remedies with herbal medicines (43.9%), turning to prayer (39.5%) or visiting a rural health clinic (31.5%). Table 7 shows the frequency of these responses according to gender and age group. The three‐way interaction between age group, gender and health‐seeking behaviour for illness treatment was not significant (*p* = 0.1689), but the two‐way interactions between age group and behaviour (*p* < 0.0001) as well as gender and behaviour (*p* < 0.0343) were significant as follows:

**TABLE 6:** Health‐seeking behaviours for illnesses (*N* = 1202).

Health seeking behaviour	Total	
	*N*	%
No Treatment	29	2.4
Herbal Medicine	528	43.9
Friends	67	5.6
Doctor	831	69.1
Traditional Healers	34	2.8
Prayer	475	39.5
Rural Health Centre	378	31.5
Other trusted people	115	9.6

**TABLE 7:** Frequency of responses on health‐seeking behaviours for illnesses according to gender and age group.

Source of information	Younger Filipinos (ages 18–59)		Older Filipinos (age ≥60)		Total	
	*N*	%	*N*	%	*N*	%
No treatment						
Male	10	3.6	1	0.7	11	2.6
Female	14	2.8	4	1.5	18	2.3
Herbal Medicine						
Male	114	40.9	64	43.5	178	41.8
Female	231	45.2	119	44.9	350	45.1
Friends						
Male	18	6.5	9	6.1	27	6.4
Female	30	5.9	10	3.8	40	5.2
Doctor						
Male	179	64.2	107	72.8	286	67.1
Female	347	67.9	198	74.7	545	70.2
Traditional Healers						
Male	7	2.5	5	3.4	12	2.8
Female	14	2.7	8	3.0	22	2.8
Prayer						
Male	107	38.4	46	31.3	153	35.9
Female	195	38.2	127	47.9	322	41.5
Rural Health Centre						
Male	77	27.6	74	50.3	151	35.5
Female	102	20.0	125	47.2	227	29.3
Other Trusted People						
Male	32	11.5	12	8.2	44	10.3
Female	58	11.4	13	4.9	71	9.2

#### 4.3.1 Age and health‐seeking behaviours for illness treatment (Tables 7 and 8)

**TABLE 8:** Odds ratio between age groups (younger/older) for health‐seeking behaviours in illness treatment.

Provider sought	Estimated odds ratio
Doctor	0.69 (1.44)**
Self‐Medication with Herbal Medicine	0.95 (1.05)
Medication from Friends	1.30 (0.77)
Rural Health Centre	0.32 (3.09)**
No Treatment	3.13 (0.32)
Prayer	0.96 (1.04)
Traditional Healers	0.81 (1.23)
Trusted People	1.90 (0.526)**

*Note*: From the generalized linear model, (**) indicates significance at the 0.01 level. Comparisons involving age groups (younger/older) use the multiplicative inverse in parenthesis.

At the 0.05 significance level, both younger and older Filipinos sought help from doctors more than all the other options, with respective, estimated probabilities of 66.13% (95% CI: 62.59, 69.49) and 73.77% (95% CI: 69.10, 77.95). This indicates that older Filipinos are 1.44 (95% CI: 1.09, 1.90) times more likely to seek a doctor than younger Filipinos (*p* = 0.0096).

Among younger Filipinos, their next choice for illness treatment after doctors is herbal medicine estimated at 43.02% (95% CI: 39.43%, 46.68%), followed by prayer at 38.25% (95% CI: 34.77%, 41.87%). Among older Filipinos, their next choice for treatment is the rural health centre estimated at 48.75% (95% CI: 43.73%, 53.81%), herbal medicine at 44.23% (95% CI: 39.27%, 49.29%) and prayer at 39.30% (95% CI: 34.36%, 44.47%). Older Filipinos are estimated to be 3.09 (95% CI: 2.37, 4.02) times more likely to go to the rural health centre for treatment than younger Filipinos.

#### 4.3.2 Gender and health‐seeking behaviours for illness treatment (Tables 7 and 9)

**TABLE 9:** Odds ratio between genders (female/male) for health‐seeking behaviours in illness treatment.

Provider sought	Estimated odds ratio
Doctor	1.15 (0.87)
Self‐Medication with Herbal Medicine	1.12 (0.89)
Medication from Friends	0.74 (1.35)
Rural Health Centre	0.76 (1.32)**
No Treatment	1.31 (0.76)
Prayer	1.42 (0.70)**
Traditional Healers	0.98 (1.02)

*Note*: From the generalized linear model, (**) indicates significance at the 0.01 level. Comparisons involving genders (female/male) use the multiplicative inverse in parenthesis.

At the 0.05 significance level, Filipino males and females sought help from doctors more than all the other options, with respective, estimated probabilities of 68.64% (95% CI: 63.73%, 73.16%) and 71.50% (95% CI: 67.97%, 74.78%). The next choice for illness treatment among females is herbal medicine at 45.05% (95% CI: 41.38%, 48.78%) and prayer at 42.97% (95% CI: 39.33%, 46.69%). The odds of a Filipino female seeking treatment through prayer is 1.42 times (95% CI: 1.09, 1.84) higher than that of a Filipino male.

The next choice of illness treatment for males after doctors is also herbal medicine estimated at 42.19% (95% CI: 37.34%, 47.20%), followed by the rural health centre at 38.33% (95% CI: 33.53%, 43.37%), and prayer at 34.74% (95% CI: 30.08%, 39.70%). The odds of a Filipino male going to a rural health centre for treatment is 1.32 times (95% CI: 1.01, 1.72) higher than that of a Filipino female.

## 5 DISCUSSION

Our findings provide new information for elucidating the sources of health information and health‐seeking behaviours of individuals living in medically underserved communities in the Philippines. We assessed the participants' health‐seeking behaviours for health promotion and treatment of an illness. Furthermore, our research compared and contrasted the information‐gathering practices and health‐seeking behaviours of both genders and age groups.

Our demographic data reflects the socioeconomic and medical vulnerability of our participants. Although there was a high number of high school graduates, most were unemployed, the majority were without insurance and most had low‐annual income (PHP 75,000 is equivalent to about $1300). The Philippines reports a high‐basic literacy rate (ability to read and write). However, many Filipinos are uninsured, with almost 1 in 5 living in poverty and having an average annual income of about $6000 (Republic of the Philippines, 2022).

### 5.1 Sources of health information

Most Filipinos in our sample relied on recommendations from family and friends to improve their health and avoid getting sick. Previous studies have also demonstrated reliance on family and friends for health information, especially among older adults (Onuegbu et al., 2021). Similarly, older adults in rural Taiwan also resorted to seeking health support from family and friends (Chen, 2020). Younger women from Senegal listed their teachers, parents or friends as their primary resources for learning about general health concerns and healthy lifestyle behaviours (Adams et al., 2017). It has been postulated that those in one's immediate social network, the people one sees and interacts with most frequently, are considered the most reliable sources of information (Teo et al., 2021). Social networks allow opportunities to exchange health information, disclose and receive treatment recommendations from those they are comfortable with, without the fear of being judged (Fiolet et al., 2021). This study supports the widespread belief that persons who live in economically and medically underserved areas are less likely to use healthcare professionals as their key source of health information, possibly due to structural barriers (such as cost or distance) and lack of access to more extensive networks beyond their immediate environment. Thus, seeking health information from family and friends is common in LIMCs. It is a way for individuals to obtain health information and practical resources to aid their health‐seeking decisions (Onuegbu et al., 2021). Our findings also support and further characterize the impact of social relationships on the health and healthcare‐seeking behaviours of people from other ethnic minority groups (Eley et al., 2019).

The second most common source of health information for the participants in our study was traditional media, a form of mass communication before the rise of digital media or the Internet. It is well established that the Internet and other social media play a significant role in spreading awareness about health issues, especially among the younger generation. However, unlike other studies that describe social media users online, our participants reported that they gathered information about health issues from traditional broadcast media such as television and radio, particularly our younger participants. Research indicates that persons in LMICs are more likely to get health‐related information traditionally without actively seeking it on online platforms, suggesting that this may be the case for our participants who do not have internet access that allows for actively searching out health information. Our findings confirm that a lack of resources and infrastructure makes it challenging for Filipinos living in medically underserved areas to obtain health‐related information to support self‐management.

A small percentage of our participants reported receiving health information from healthcare professionals, and younger participants were more likely to fall into this category than older participants. These findings align with reports that more healthcare consumers rely on other sources for health information, particularly older adults. Since the participants in our study were treated inside a public healthcare system with considerable time constraints, they may have had limited time for one‐on‐one interactions with their healthcare professionals. In addition, many people may not have had access to a healthcare professional requiring them to seek health information elsewhere. This may result in inaccurate health information being communicated by untrained individuals and the inability of patients to receive scientifically factual and evidence‐based answers to health concerns (Bitton et al., 2019). Similarly, we might hypothesize that the prevalence of a public's need for health‐related information is influenced by the attitude of consumers about healthcare professionals' training, education and skills. As a result of a potential lack of confidence in the trustworthiness of their healthcare professionals, it is typical for individuals to seek information elsewhere for their healthcare needs (Lodenstein et al., 2017).

### 5.2 Provider sought for health promotion

Health promotion is a process that enables individuals to take greater responsibility for and improve their health. The fundamental goals of health promotion are disease prevention and enhancing an individual's ability for self‐care (Mehri et al., 2016). Participating in health‐improving behaviours is one of the most crucial parts of disease prevention. These efforts not only lower the likelihood of disease but also enhance the health and longevity of patients. In contrast, failure to adopt these habits is connected with the development of diseases and higher mortality (Buse et al., 2017).

One of the aims of the current study was to examine the health‐seeking behaviours of Filipinos living in medically underserved regions of the country related to health promotion. Our findings showed that about 61% of the participants sought help for health promotion from healthcare professionals. This number may reflect health promotion services being increasingly free in local government and rural health clinics. However, this still leaves a good number of the population who are not seeking health professionals for health promotion, even when free in some circumstances, which is likely related to our prior finding that participants minimally received health information from healthcare professionals as their primary source.

Less than a fifth of our participants sought help from informal healthcare providers. In other studies, such as those conducted among Korean immigrants in the United States, fear of American doctors and western medicine led some to seek alternative treatments. Some people also felt that asking for assistance was useless because of bad luck, sin, karma or fate (Chung et al., 2018). Thus, appealing to traditional customs and indigenous knowledge by seeking health information from informal healthcare providers is not unusual in these cases. Researchers urge that such acceptable health promotion methods be examined to satisfy the health information needs of adults in medically underserved communities in light of issues including lack of infrastructure, poverty and high‐illiteracy rates.

In the Philippines, there are several types of informal healthcare providers. For example, the herbalist, also known as an albularyo, is an unlicensed general practitioner experienced in providing folkloric medicines and well‐versed in treating their clients with medical herbal remedies. In addition, manghihilots or hilots provide treatments for sprains, fractures and any musculoskeletal concerns, similar to massage therapists or chiropractors, except they do not receive formal or professional training (Rebuya et al., 2020). Finally, faith healers or espiritistas, use spiritual energies through religion, supernatural interventions or faith to treat their clients' physical, mental and spiritual concerns. Our findings indicate that about 1 in 5 still seek informal healthcare providers for health promotion. Effectively integrating these providers into the healthcare system and providing them with adequate training and education regarding health promotion is important because such providers allow access to healthcare that would not have been possible otherwise, and they remain an integral part of the Philippine culture, especially in areas far from urban regions (Rebuya et al., 2020).

Almost 1 in 10 of our participants sought a combination of providers (professionals and informal providers) while others may not have accessed health promotion services until the need arises for illness treatment. Although we did not find any gender or age interaction in our research, previous studies of older women in LMICs showed that in comparison to younger women and men, older women were less likely to be proactive in participating in prevention screening and health promotion programs (Debesay et al., 2022). Lack of time and energy to focus on one's health due to a combination of family and local obligations and potentially opposing viewpoints on the severity of various health conditions and the importance of preventive healthcare is likely to account for these findings. Efforts to recognize potential health disparities should support health promotion and healthy lifestyle behaviours.

Health promotion initiatives should encourage multiple sources of evidence‐based health information and healthcare professionals, including public health nurses and trained *barangay* health workers, targeted to the needs and concerns of medically underserved individuals. For instance, health clinics could help provide necessary information through brochures, *barangay* health sessions or health fairs that detail disease prevention and wellness promotion activities and symptoms and treatment options for various diseases. Health education programs delivered by well‐trained *barangay* health workers may be an efficient and accessible source of knowledge regarding general health topics and health promotion practices supported and maintained by public health nurses in rural health centres. Having wellness checks and regular check‐ups from healthcare professionals are encouraged for health promotion and disease prevention while allowing for individualized and targeted health information based on specific needs, particularly those having more specialized needs beyond general health information.

### 5.3 Health‐seeking behaviours for illness treatment

Only at least two‐thirds of our participants reported seeing a doctor when they recognized symptoms of being sick, while much less (almost one‐third) visited the rural health clinic. Research shows that individuals from LMICs reported experiencing multiple barriers when seeking healthcare professionals. The key challenges to care in these studies were lack of insurance, transportation, the expense of care, lack of information on available services, long wait times for appointments and limited hours of operation (Maneze et al., 2015). Because our participants resided in medically underserved communities, it is reasonable to presume that these barriers also existed. Almost half of our participants used self‐medication (i.e., herbal medicines) as home remedies while others turned to prayer when ill. These could also be related to preferences for spirituality, self‐management and resilience despite an illness, commonly reported among many Asians (Kawi et al., 2019).

Our findings also confer that older Filipino adults were more likely to visit a doctor when sick; some needed medical attention and went to the nearest health clinic compared to younger adults. This difference in health‐seeking behaviour by age corroborates findings from other research on age‐related differences in health‐related behaviour. Older persons, for example, have been found to have a higher level of consciousness and awareness, a greater sense of responsibility for their health and, inevitably, a greater level of concern regarding their health. However, a recent scoping review of 52 studies on the health‐seeking behaviours of older adults showed contrasting findings; that older adults often failed to ask for help when ill (Teo et al., 2021). In addition, the review reported that older adults would commonly self‐assess their health first to determine whether seeking help was necessary; they often indicated that health‐seeking was viewed as a threat to their independence.

Not rated highly in our study findings were participants seeking illness treatment from informal healthcare providers (i.e., traditional healers). However, in other LMIC studies, seeking treatment from traditional healers was not unusual because causal beliefs influenced the use of informal healthcare providers regarding symptoms, family pressure to see an informal healthcare provider, convenience, price and a desire to shun conventional healthcare (McCutchan et al., 2021).

Concerning age and health‐seeking behaviours for illness treatment, apart from seeing doctors, our findings did not show significant differences in age in using other modalities such as herbal medicines or prayer. In other studies, older adults were more likely to use nonprescribed therapies to treat or prevent illness (Arcury et al., 2015). Another study that measured non‐prescribed therapy for managing an illness showed that over 65% of older adults reported utilizing at least one non‐prescribed therapy specifically for illness prevention. The most popular treatment was prayer (80.7%), followed by over‐the‐counter drugs (54.3%), vitamins solely (49.3%), herbs and supplements (40.5%), physical activity (31.9%) and home cures (5.2%) (Altizer et al., 2014).

In relation to gender and health‐seeking behaviours for treatment, we did not find significant differences in using other modalities other than seeking doctors such as herbal medicines. However, we found that Filipino women are more likely to use prayer than men, while men are more likely to seek the rural health centre for treatment than women. Previous research showed that men were more likely to seek healthcare services from healthcare professionals (e.g., in health centres), while women tended to favour alternative treatment (Das et al., 2018). For example, women of Hispanic and Asian ancestry were more likely to utilize nonprescribed therapies to treat or prevent illness (Arcury et al., 2015). Women in our study may have faced more social barriers to seeking formal healthcare support, which may explain why they were more likely to turn to prayer or alternative remedies. Healthcare is one area where women's traditionally lower social status has led to disadvantages (Das et al., 2018). Women also experience more burdens of cultural expectations, social obligations and financial load than men. In contrast to men, women report a greater variety of reasons for adopting therapeutic alternatives associated with adjusting to these circumstances (Patra & Bandyopadhyay, 2020). This may be crucial when determining whether women receive adequate healthcare relative to men. The underlying causes of the seemingly contradictory or, at the very least, misconstrued complementary nature of healthcare professionals and alternative healthcare services require additional exploration (Das et al., 2018).

A contrasting finding was reported in a study involving African American and Latino men with type 2 diabetes revealing that men's ideas about manhood and the need to maintain control over their health (e.g., maintaining a “strong image” and being reluctant to heed health advice) inhibited their health‐seeking behaviours (Eley et al., 2019). The effects of these beliefs can be seen in reduced use of healthcare services, delayed response to symptoms, nonadherence to prescribed medications and reluctance to discuss health problems openly.

Given the differences in study designs and samples analysed in existing literature, it is not surprising that the current state of science exhibits variable findings (Alegana et al., 2017). Nonetheless, disparities in healthcare‐seeking behaviours by age and gender are likely to affect overall wellness. For instance, they might be responsible for a sizable amount of the continuing gap in life expectancy between the genders. Consequently, everyone would benefit from an efficient strategy to advance age and gender parity in healthcare for easier access to healthcare professionals before making important healthcare decisions for medical treatment. Therefore, public health initiatives and treatments must be age‐ and gender‐sensitive and consider various approaches (Fareed et al., 2021), particularly to help address the needs of medically underserved populations.

## 6 STRENGTHS AND LIMITATIONS

Little research has been conducted in the Philippines on where people access and receive health information or what they do when they require healthcare services for health promotion and treatment. The key strength of this study is the use of a very large population‐based representative sample to examine health information sources and health‐seeking behaviours, thereby maximizing the study's ability to detect significant age and gender disparities. Moreover, because so little is known about how medically underserved individuals utilize healthcare, we focused on this vulnerable population in our research. This study also investigated the impact of age and gender on health‐seeking behaviours.

Our study has limitations. Participants were selected from their communities, some of which lacked access to a rural health facility within 30 min of their location. As a result, they may not be representative of those who live in areas with better access to local, provincial or regional healthcare facilities, where access to health information may be higher. However, the high proportion of unemployed and uninsured participants suggests that our study may have captured those most at risk of experiencing health disparities. In addition, the daily stress experienced by those in low‐income communities likely leads to delays in seeking healthcare.

Likewise, specific healthcare services were not evaluated, including maternity and paediatric healthcare utilization. Further, we did not collect information on how individuals utilized the received health‐related information, so we do not know if seeking health‐related information improved decision‐making. Finally, as a cross‐sectional study, we are limited in the conclusions we can draw regarding causation. Although there were some limitations with the study's methodology, they were deemed to be outweighed by the significance of investigating a topic where few studies have been conducted, particularly among a large number of individuals in underserved communities in a developing country.

## 7 PRACTICE AND RESEARCH IMPLICATIONS

Some of our results were consistent with previous studies, while others were contrasting. Either way, most countries must fight to make healthcare more accessible and widespread in underserved communities specific to their needs. Increased internet penetration and the availability of cable and satellite television services are altering the media landscape in previously medically underserved areas of LMICs, raising new questions about how certain populations in the Philippines consume and react to news and other forms of media. People from marginalized groups need to be able to pursue and gain access to information actively. In addition, academics and policymakers should investigate how rural communities can benefit from broader access to credible health information and better target activities to offer such information. Additional key factors include age, gender, income, distance to the rural health centre, medical conditions and perceived disease severity.

Our findings showed that only 61% of individuals in medically underserved areas of the Philippines sought healthcare professionals for wellness, and only 69% visited a physician when they were ill. This leaves about one‐third of individuals who do not utilize healthcare professionals when needed. Many resort to alternative modalities such as herbal medicines and prayer and rely on their social networks as their key source for health information instead of healthcare professionals. In addition, we did not see a high utilization of informal healthcare providers (e.g., traditional healers). Investigation of sources of health information and health‐seeking behaviours continue to require further examination for better clarity to inform health programs and policy.

Similarly, the hurdles that prevent this vulnerable population from obtaining professional healthcare require additional investigation. Age and gender were also significantly associated with health‐seeking behaviours; researchers and clinicians must intricately examine particular subgroups and their risks of receiving suboptimal healthcare services. The outcomes of this study emphasize the necessity for addressing health‐seeking behaviours as well as age and gender when designing health promotion campaigns, activities and intervention programs. Public health nurses figure prominently in these strategies.

Finally, our findings suggest that more national research should be done. Meanwhile, local government units in the nation's medically underserved regions should continue to address issues such as access to healthcare professionals, including public health nurses, healthcare inequality, lack of investment in healthcare infrastructure and the inaccessibility of clinics in remote places. By focusing on and overcoming these concerns, healthcare could be enhanced. Likewise, Filipinos in medically underserved areas' health literacy and collaborative decision‐making practices should be studied in the future. Additional research is warranted to discover whether individuals have conducted an information search related to their health, the channels used, the appraisal of information gained and how they acted on the health information they received.

## 8 CONCLUSION

Understanding sources of health information and health‐seeking behaviours is essential for developing healthcare policies and programs, as it identifies potential barriers to early diagnosis, facilitates implementation of effective interventions and can facilitate successful treatment. It is possible to improve adherence to plans of care, including proper medications, and reduce morbidity and mortality through accurate information as well as early diagnosis and treatment by healthcare professionals. Policymakers in LMICs, like in the Philippines, need to consider the medically underserved population's health‐seeking behaviours and access to health‐related information from the most credible sources when designing health education and disease prevention programs. Increasing internet access in developing countries presents a window of opportunity for online initiatives, but other approaches may be needed to reach specific populations. Health information should be shared using multiple platforms, including more popular and accessible sources such as television and radio. These may also include gender‐ and age‐specific approaches that address the unique interests and concerns of people in medically underserved areas.

## AUTHOR CONTRIBUTIONS

Jennifer Kawi: Conceptualization (co‐lead), data curation, methodology (co‐lead), validation, writing, review and editing. Miguel Fudolig: Conceptualization, data curation, formal analysis, methodology, writing, review and editing. Reimund Serafica: Conceptualization, writing, review and editing. Andrew Reyes: Conceptualization, writing, review and editing. Francisco Sy: Conceptualization, validation, writing, review and editing. Erwin Leyva: Conceptualization, investigation, resources. Lorraine Evangelista: Conceptualization (lead), data curation, formal analysis, investigation, methodology (lead), resources, supervision, validation, writing, review and editing.

## ACKNOWLEDGEMENTS

We extend our deepest gratitude to our participants and hardworking individuals particularly in the Philippines who helped in one way or the other to help facilitate the completion of our parent study.

## FUNDING INFORMATION

This research received no specific grant from any funding agency in the public, commercial or not‐for‐profit sectors.

## CONFLICT OF INTEREST STATEMENT

The authors have no conflict of interest to declare.

## ETHICS STATEMENT

The Institutional Review Boards at a western university in the United States and a northern university in the Philippines (Research Ethics Board) approved the study. Informed consents were completed.

## STATISTICS

There is a statistician in the author team (Miguel Fudolig).

## Data Availability

The data that support the findings of this study are available from the corresponding author, (Jennifer Kawi), upon reasonable request
